# The efficacy of self-management programmes for increasing physical activity in community-dwelling adults with acquired brain injury (ABI): a systematic review

**DOI:** 10.1186/2046-4053-3-39

**Published:** 2014-04-21

**Authors:** Taryn M Jones, Julia M Hush, Blake F Dear, Nickolai Titov, Catherine M Dean

**Affiliations:** 1 Department of Health Professions, Faculty of Human Sciences, Macquarie University, Ground Floor, 75 Talavera Rd, 2109 Sydney, NSW, Australia; 2 Centre for Emotional Health, Department of Psychology, Faculty of Human Sciences, Macquarie University, 2109 Sydney, NSW, Australia; 3 Centre for Physical Health, Faculty of Human Sciences, Macquarie University, Sydney, NSW, Australia

**Keywords:** Self-management, Physical activity, Brain injury, Stroke, Remote delivery, Internet

## Abstract

**Background:**

Acquired brain injury (ABI), often arising from stroke or trauma, is a common cause of long-term disability, physical inactivity and poor health outcomes globally. Individuals with ABI face many barriers to increasing physical activity, such as impaired mobility, access to services and knowledge regarding management of physical activity. Self-management programmes aim to build skills to enable an individual to manage their condition, including their physical activity levels, over a long period of time. Programme delivery modes can include traditional face-to-face methods, or remote delivery, such as via the Internet. However, it is unknown how effective these programmes are at specifically improving physical activity in community-dwelling adults with ABI, or how effective and acceptable remote delivery of self-management programmes is for this population.

**Methods/Design:**

We will conduct a comprehensive search for articles indexed on MEDLINE, EMBASE, CINAHL, PsychINFO, AMED, Cochrane Central Register of Controlled Trials (CENTRAL), PEDro and Science Citation Index Expanded (SCI-EXPANDED) databases that assess the efficacy of a self-management intervention, which aims to enhance levels of physical activity in adults living in the community with ABI. Two independent reviewers will screen studies for eligibility, assess risk of bias, and extract relevant data. Where possible, a meta-analysis will be performed to calculate the overall effect size of self-management interventions on physical activity levels and on outcomes associated with physical activity. A comparison will also be made between face-to-face and remote delivery modes of self-management programmes, in order to examine efficacy and acceptability. A content analysis of self-management programmes will also be conducted to compare aspects of the intervention that are associated with more favourable outcomes.

**Discussion:**

This systematic review aims to review the efficacy of self-management programmes aimed at increasing physical activity levels in adults living in the community with ABI, and the efficacy and acceptability of remote delivery of these programmes. If effective, remote delivery of self-management programmes may offer an alternative way to overcome barriers and empower individuals with ABI to increase their levels of physical activity, improving health and general wellbeing.

**Trial registration:**

Our protocol has been registered on PROSPERO 2013: CRD42013006748.

## Background

Physical inactivity is globally recognised as a major cause of morbidity and mortality. Physical inactivity is now identified as the fourth leading risk factor for global mortality with levels of inactivity rising in most countries [1]. Acquired brain injury (ABI) is a significant cause of morbidity and burden globally, leading to significantly reduced levels of physical activity. Individuals with ABI suffer reduced health and wellbeing as a result of being inactive, increasing the global burden of non-communicative disease (NCD) caused by physical inactivity [2-4].

ABI refers to any damage to the brain that occurs after birth. The most common cause of ABI is stroke or trauma. Stroke is the second most common cause of mortality worldwide, while traumatic brain injury (TBI) is the leading cause of death and disability in children and young adults around the world [1]. ABI is a major cause of disability - stroke alone accounts for a loss of 49 million disability-adjusted life years (DALYs) worldwide annually [1]. Disability directly reduces physical activity levels, causing a spiral of deteriorating health and quality of life in those affected by ABI. People with ABI report more disability and more health disorders than the average person with disability [3]. Almost half (46%) of people with severe or profound disability have poor health status, compared to only 5% of those without disability [4]. An Australian report indicates that one in 45 Australians has an ABI with activity limitations or participation restrictions, and that these individuals are substantially less active than those without [3].

Physical inactivity both causes and accelerates chronic disease. Rising levels of physical inactivity have major implications for the general health of people worldwide and for the prevalence of NCDs such as cardiovascular disease, diabetes and cancer, and their risk factors such as raised blood pressure, raised blood sugar and obesity [5]. It is currently estimated that six out of every 10 deaths globally are attributable to non-communicable conditions [6]. These conditions resulting from physical inactivity have very high societal burden, causing considerable morbidity and mortality [7]. Those already affected by disability, such as individuals with ABI, are at significantly greater risk of developing further chronic health conditions due to physical inactivity [3].

Despite the heightened risk of chronic health conditions in this population, services to help people living in the community with ABI increase their levels of physical activity are very limited. In Australia for example, over 50% of stroke survivors report being dissatisfied with their access to information about stroke recovery, as well as being frustrated in trying to determine what services are available, where they are located, and whether they meet their personal circumstances [8]. There are a number of common barriers, such as direct and indirect costs of treatment, transport difficulties and limited local specialist services [8-10], which significantly reduce the uptake of physical activity among individuals with ABI. In developing countries, the situation is even worse with a substantial lack of resources, funding and knowledge available to assist those with disability resulting from ABI [2].

The challenge of sustaining physical activity is also often complicated in people with ABI because the impact of their condition changes over time, especially as individuals enter different stages of their life. In Australia, 75% of individuals with an ABI are aged under 65 years, and two out of three of these individuals are aged under 25 years [11]. Thus, many people are left to manage their physical activity levels over a long period of their life and encounter different challenges at different times. For example, an individual may alter their employment status or start a family. Life changes, such as these, can present different challenges to physical activity. In order to best sustain physical activity in the long term it is imperative that individuals living with ABI be empowered with adequate knowledge and self-management skills to adapt to changing barriers. Self-management skills, such as problem-solving, decision making and resource utilisation, are paramount to building self-efficacy and enabling individuals to make informed choices in managing their health over their lifespan [12].

The World Health Organization (WHO) has estimated that by 2020, chronic disease will account for 75% of all deaths globally [13]. The WHO has argued for nations to do more to prevent chronic disease [7], particularly through the introduction of strategies to increase physical activity [1]. In response, the Australian Government developed the National Chronic Disease Strategy (NCDS) of which a key focus is self-management. The NCDS emphasises the importance of tailoring self-management approaches to the unique needs of different disease populations in order to improve uptake of physical activity. Information needs to be delivered in an appropriate format for people to comprehend and people must be offered approaches that are personally relevant, yet evidence-based [13]. There is considerable evidence that self-management programmes result in better long-term outcomes for people with chronic diseases [14-16]. This includes programmes for individuals with ABI, specifically stroke [17,18]. Despite this, many people with ABI do not receive self-management training. In the National Stroke Audit undertaken in Australia in 2012, only 25% of stroke survivors were informed about self-management programmes, a decline from 40% in 2008 [19].

The mode of self-management programme delivery can alter the scope of access. Compared with face-to-face delivery, remote delivery modes, such as the Internet, may increase accessibility for those who face multiple barriers to accessing optimal healthcare [20], such as cost, mobility restrictions or service availability in rural or remote regions. Delivery of self-management programmes via the Internet has been used with success in a variety of populations, such as chronic pain [21], anxiety and depression [22], post-traumatic stress disorder [23], arthritis [24] and cerebral palsy [25]. The potential for remote-based delivery methods to be utilised to increase physical activity has also been reported by Foster and colleagues in a recent Cochrane review [26]. However, to date, there has been no systematic review of the literature examining the efficacy of self-management programmes on physical activity for individuals with ABI.

The aim of this systematic review is to address this knowledge gap. We will conduct a systematic review to investigate the efficacy of self-management programmes on physical activity specifically in individuals with ABI. We aim to answer the following questions:

1. How effective are self-management programmes in improving physical activity in community-dwelling adults with ABI?

2. How effective and acceptable is remote delivery of self-management programmes aimed at improving physical activity in community-dwelling adults with ABI?

3. Which features of self-management programmes aimed at improving physical activity in community-dwelling adults with ABI are associated with the best clinical outcomes and client satisfaction?

## Methods/Design

### Study registration

The protocol of this systematic review has been registered on PROSPERO 2013 (registration number: CRD42013006748) [27]. The systematic review protocol has been conducted and reported using the Preferred Reporting Items for Systematic Reviews and Meta-Analyses (PRISMA) statement guidelines [28].

### Search strategy for identification of relevant studies

We will conduct a comprehensive search for articles indexed on MEDLINE, EMBASE, CINAHL, PsycINFO, AMED, Cochrane Central Register of Controlled Trials (CENTRAL), PEDro and Science Citation Index Expanded (SCI-EXPANDED) databases from their inception to December 2013. A search strategy has been designed with the assistance of an experienced research librarian. We have developed a search strategy in MEDLINE (Appendix 1) which has been customised to account for differences in indexing across other databases. We will screen the reference lists of relevant reviews on this topic to identify further studies for potential inclusion in this review. Non-English language studies will be included, where a translation can be made available.

### Eligibility criteria

#### Types of study

We will include only those studies that are randomised controlled trials, or quasi-randomised controlled trials. A quasi-randomised controlled trial is defined as a trial in which the participant’s allocation is not truly random, such an allocation by date of birth.

#### Participants

We will include studies of adults (aged 18 years or older) living in the community who have a non-degenerative ABI. ABI refers to any damage to the brain that occurred after birth, such as from trauma or a stroke. We will exclude any studies that examine ABI that is degenerative in nature, such as studies of Parkinson’s disease, or studies of people undergoing significant medical or surgical intervention, such as chemotherapy. However, participants who have sustained an ABI as a result of an adverse outcome from a surgery will be included. We will also exclude papers where this status is unclear, such as various types of brain cancer. We will also exclude any studies of people residing in nursing homes or other non-independent care facilities, or who are inpatients in a hospital or other healthcare facility. There will be no restriction of duration since injury.

In studies where it is unclear that participants meet our inclusion criteria we will contact the study author for verification. We will exclude any studies where verification cannot be made by the author in regards to our inclusion criteria. Studies in which there is a mixed sample (with respect to residential status, age or health condition) will only be included if at least 75% of the participants meet our inclusion criteria.

#### Intervention

We will include studies that have assessed the efficacy of a self-management intervention which aims to enhance levels of physical activity or other outcomes specifically associated with physical activity. Physical activity refers to any bodily movement produced by skeletal muscles that requires energy expenditure [29]. Other outcomes associated with physical activity include physical activity related self-efficacy, physical self-concept, social support or decisional balance for physical activity, and stages of change in regards to physical activity.

Interventions can be provided by health professionals, lay people or a combination of both. Interventions can be delivered in a group setting or on an individual basis. The self-management intervention may be generic or specific to a health condition; however it must include at least one of the following components: problem-solving, goal-setting, decision-making, self-monitoring, coping strategies or another approach to facilitate behaviour change. Studies including advice and education only will be excluded.

The self-management programme can be administered in a variety of settings, such as a private home, a hospital or a community centre. However, participants must be community-dwelling.

For review question 1, the intervention may be delivered via a variety of delivery formats, such as face-to-face, text messages, telephone, Internet or postal delivery. For review question 2, the intervention will include only those studies that have self-management programmes delivered remotely, such as via the Internet, text messages, telephone or by postal delivery. Any studies that have directly compared two types of self-management approaches will be included for content analysis.

#### Comparator or control

For review question 1, we will include studies that compare a self-management intervention with any of the following: usual care, waiting list control, no treatment, written information only, education and advice only, or an alternative treatment that is not considered to be self-management. For review question 2, comparative studies will be those papers that met all the inclusion criteria for review question 1, and delivered the self-management programme via face-to-face delivery. As stated above, any studies that have directly compared two types of self-management approaches will be included for content analysis.

### Outcome measures

#### Primary outcomes

We will include studies that have examined at least one of the following primary outcome measures:

● A measure of physical activity, either from a physical activity monitoring device (for example, accelerometer, pedometer), or from a self-report measure of physical activity; and/or;

● A primary study outcome associated specifically with physical activity; such as physical activity self-efficacy or physical self-concept.

We will extract data for primary outcomes assessed at baseline and all follow-up time points.

#### Secondary outcomes

For studies that meet inclusion criteria the following secondary outcomes will also be examined:

● Self-efficacy (general) - usually measured by a self-efficacy scale, such as the General Self-Efficacy Scale [30] or the Stroke Self-Efficacy Scale [31];

● Participation measures - such as the Modified Reintegration to Normal Living Index (mRNL) [32], Life Habits questionnaire (LIFE-H) [33] or the Community Integration Questionnaire (CIQ) [34];

● Activity measures - such as the Step test, 10 m walk test , 6 min walk test, Timed Up and Go test;

● Impairments - such as depression (for example, PHQ-9 [35]), anxiety (for example, GAD-7 [36]), strength, cardiovascular fitness;

● Quality of life measures - such as the WHO Disability Assessment Schedule (WHODAS-II) [37], or the WHO Quality of Life assessment instrument (WHOQoL) [38];

● Participant satisfaction - either quantitative or qualitative;

● Cost-effectiveness.

We will extract data for secondary outcomes assessed at baseline and all follow-up time points.

We will also record any adverse outcomes that are reported in studies included in this review.

### Screening of studies

Studies will be selected for this review by two authors who will independently assess the titles and abstracts of all records identified from the searches of the electronic databases by excluding studies that do not meet all inclusion criteria. The full text of the remaining studies will be obtained and independently reviewed by two authors for eligibility according to the criteria using a standardised eligibility criteria sheet. If needed, further information will be obtained from the authors where possible. Disagreements will be resolved by discussion and consensus. If required, arbitration will occur by a third review author blinded to previous eligibility ratings.

### Data extraction

Data from the remaining included studies will be extracted independently by two reviewers using a standardized data extraction form. This form will include collection of the following data: source, year of publication, country of origin, study design, sample size (including participants that have been lost to follow-up), characteristics of the study population (age, gender and cause of ABI), characteristics of the intervention (delivery mode and method, duration, description of content), characteristics of control/comparison (delivery mode, duration, description of content), type of outcome measures used - primary and secondary, outcome measures for identified time points as above and statistical analysis.

### Risk of bias (quality) assessment

Two reviewers will independently assess the risk of bias for each included study using *The Cochrane Collaboration’s tool for assessing bias*[39]. The criteria included in this tool are: random sequence generation, concealed allocation, blinding, completeness of data collection and selective outcome reporting. We will summarise bias as being ‘low’, ‘high’ or ‘unclear’ for each criterion. A summary of risk of bias across all studies within each domain will also be provided.

### Strategy for data synthesis

For review questions 1 and 2, Review Manager software, *RevMan*[40], will be used to conduct a meta-analysis where possible to calculate an overall effect size for physical activity. Where data are too heterogeneous to perform a meta-analysis, the results from individual studies will be summarised in a table and a narrative synthesis will be conducted. If, during this synthesis, homogeneity is established within a subgroup, a meta-analysis of data for this subgroup will be performed. A risk of bias assessment of included studies will be summarised in a table and results and implications will be critically discussed.

In order to examine the features associated with greater efficacy and participant satisfaction, a content analysis will be conducted to compare aspects of the intervention that are associated with more favourable study outcomes.

### Analysis of subgroups or subsets

In order to address review question 2, a subgroup analysis of different mechanisms of intervention delivery will be conducted, where appropriate, to enable a comparison of remote delivery methods with traditional face to face methods of delivery.

If appropriate a subgroup analysis may be conducted to compare efficacy of self-management programmes to enhance physical activity in young adults (aged 18–50 years) *versus* older adults (aged over 50 years), and in stroke *versus* traumatic brain injury.

## Discussion

This review will examine the efficacy of self-management programmes for increasing physical activity specifically in adults living in the community with ABI. We will also examine the efficacy of remote delivery in comparison to traditional face-to-face methods. With physical inactivity being a significant cause of global morbidity and mortality it is important that effective, sustainable strategies for increasing physical activity in high-risk populations, such as those with ABI, are implemented in a manner that enhances accessibility and uptake.

## Appendix 1

### Medline search strategy

1. exp Self Care/

2. exp health education/or exp patient education as topic/

3. exp consumer participation/or exp patient participation/

4. exp health communication/or exp health promotion/

5. exp Self Concept/or exp Self Efficacy/

6. (self adj care*).mp.

7. (self adj manage*).mp.

8. (patient adj educat*).mp.

9. (self adj monitor*).mp.

10. (self adj efficacy).mp.

11. (self adj concept).mp.

12. ((consumer or patient) adj participat*).mp.

13. ((consumer or patient) adj inform*).mp.

14. (health adj educat*).mp.

15. (health adj promot*).mp.

16. 1 or 2 or 3 or 4 or 5 or 6 or 7 or 8 or 9 or 10 or 11 or 12 or 13 or 14 or 15

17. exp Motor Activity/

18. exp “activities of daily living”/or exp leisure activities/or exp recreation/

19. exp gait/or exp locomotion/or exp walking/

20. exp sports/or exp physical fitness/

21. exp Exercise/or exp Exercise Therapy/

22. exp Health Behavior/

23. (physical adj activity).mp.

24. (leisure or recreation*).mp.

25. (sport* or fit* or exercis*).mp.

26. (walk* or ambulat* or mobil* or locomotion or gait).mp.

27. 17 or 18 or 19 or 20 or 21 or 22 or 23 or 24 or 25 or 26

28. 16 and 27

29. exp brain damage, chronic/or exp brain injuries/ or exp cerebrovascular disorders/

30. (brain adj (injur* or damage)).mp.

31. stroke*.mp.

32. (cerebrovascular adj accident*).mp.

33. exp Stroke/

34. 29 or 30 or 31 or 32 or 33

35. 28 and 34

36. Randomized controlled trial.pt.

37. random*.mp.

38. trial*.mp.

39. control*.mp.

40. controlled clinical trial.pt.

41. placebo*.mp.

42. (intervention adj group*).mp.

43. (treatment adj group*).mp.

44. 36 or 37 or 38 or 39 or 40 or 41 or 42 or 43

45. 35 and 44

46. limit 45 to humans

47. remove duplicates from 46

## Competing interests

The authors declare that they have no competing interests.

## Authors’ contributions

TMJ: conception and design, data collection, analysis and interpretation, manuscript writing and final approval of manuscript. JMH: conception and design, data analysis and interpretation, manuscript writing and critical revision, and final approval of manuscript. BFD: conception and design, data analysis and interpretation, critical revision of the manuscript, and final approval of manuscript. NT: conception and design, interpretation of data, critical revision of the manuscript, and final approval of manuscript. CMD: conception and design, data collection, analysis and interpretation, manuscript writing and critical revision, and final approval of manuscript. All authors read and approved the final manuscript.
